# Supplementary material for: Evolution of beta-lactam resistance causes fitness reductions and several cases of collateral sensitivities in the human pathogen Haemophilus influenzae
Source: Antimicrob Agents Chemother. 2025 Sep 22;69(11):e00576-25. doi: 10.1128/aac.00576-25 (PMC12587571; doi:10.1128/aac.00576-25)
Supplement: Fig. S1 — Graphical protocol of the performed multi-step evolution experiment. [file aac.00576-25-s0001.pdf]

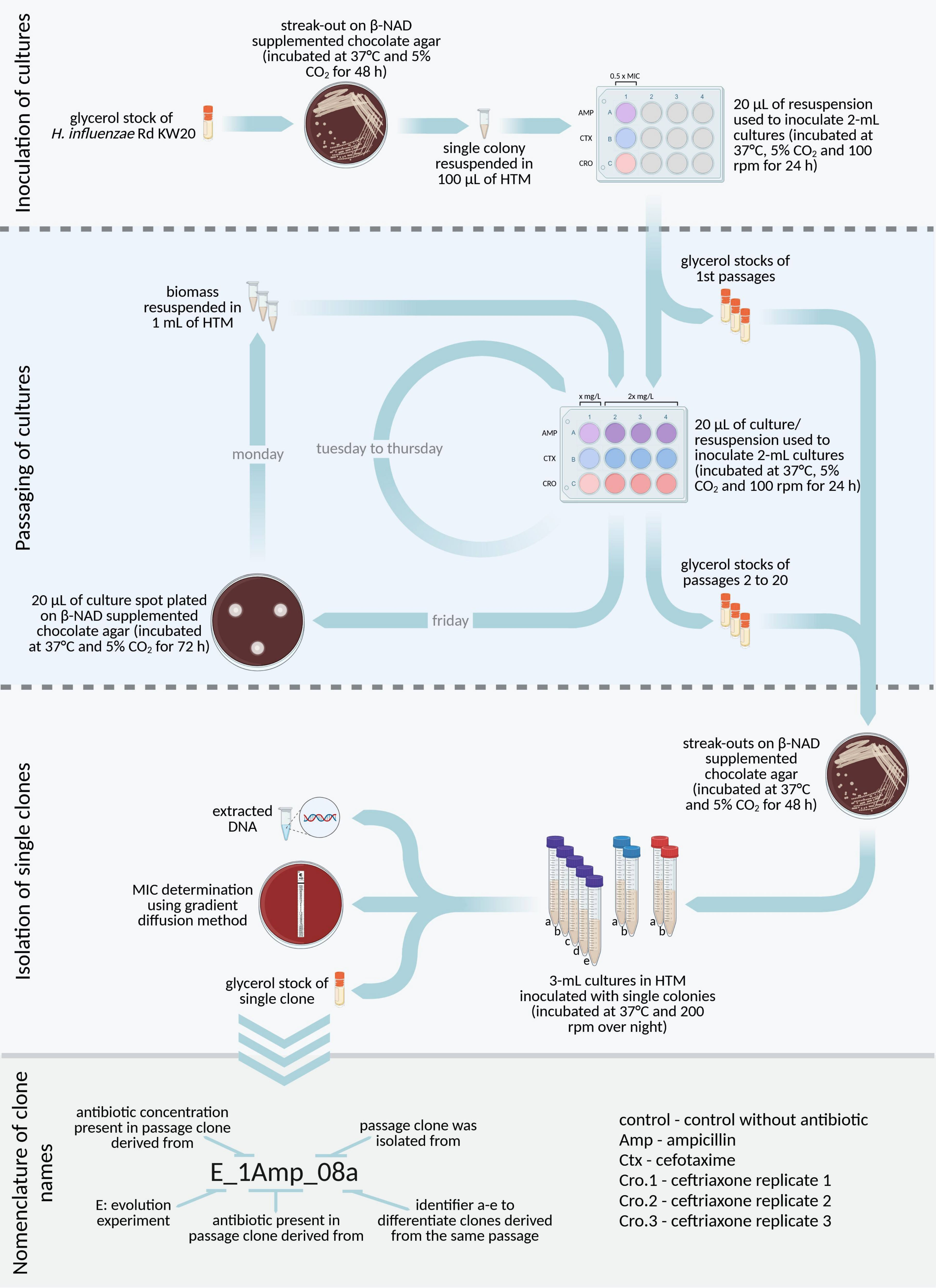

Inoculation of cultures
glycerol stock of
H. influenzae Rd KW20
streak-out on β-NAD supplemented chocolate agar (incubated at 37°C and 5% CO_2 for 48 h)
single colony resuspended in 100 µL of HTM
0.5 x MIC
1
2
3
4
AMP
CTX
CRO
A
B
C
20 µL of resuspension used to inoculate 2-mL cultures (incubated at 37°C, 5% CO_2 and 100 rpm for 24 h)
Passaging of cultures
glycerol stocks of 1st passages
biomass resuspended in 1 mL of HTM
monday
tuesday to thursday
x mg/L
2x mg/L
20 µL of culture/ resuspension used to inoculate 2-mL cultures (incubated at 37°C, 5% CO_2 and 100 rpm for 24 h)
glycerol stocks of passages 2 to 20
20 µL of culture spot plated on β-NAD supplemented chocolate agar (incubated at 37°C and 5% CO_2 for 72 h)
friday
Isolation of single clones
streak-outs on β-NAD supplemented chocolate agar (incubated at 37°C and 5% CO_2 for 48 h)
extracted DNA
MIC determination using gradient diffusion method
glycerol stock of single clone
a
b
c
d
e
a
b
a
b
3-mL cultures in HTM inoculated with single colonies (incubated at 37°C and 200 rpm over night)
Nomenclature of clone names
antibiotic concentration present in passage clone derived from
passage clone was isolated from
E_1Amp_08a
E: evolution experiment
antibiotic present in passage clone derived from
identifier a-e to differentiate clones derived from the same passage
control - control without antibiotic
Amp - ampicillin
Ctx - cefotaxime
Cro.1 - ceftriaxone replicate 1
Cro.2 - ceftriaxone replicate 2
Cro.3 - ceftriaxone replicate 3
