# Supplementary figures and images for: Evolution of beta-lactam resistance causes fitness reductions and several cases of collateral sensitivities in the human pathogen Haemophilus influenzae
Source: Antimicrob Agents Chemother. 2025 Sep 22;69(11):e00576-25. doi: 10.1128/aac.00576-25 (PMC12587571; doi:10.1128/aac.00576-25)

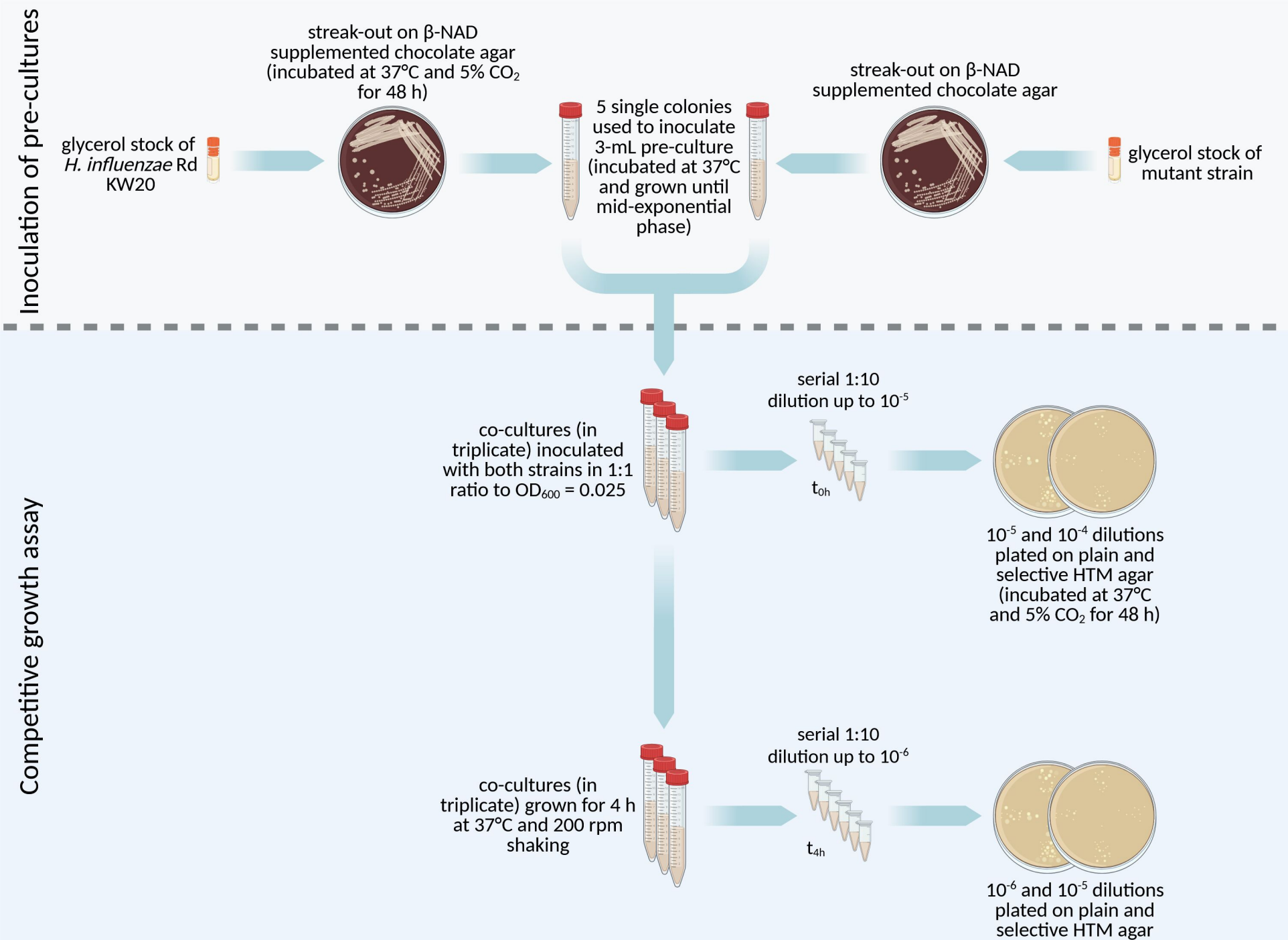

Supplement: Fig. S2 — Graphical protocol of the performed competitive growth assay. [file aac.00576-25-s0002.pdf]

A

Tree scale: 0.01

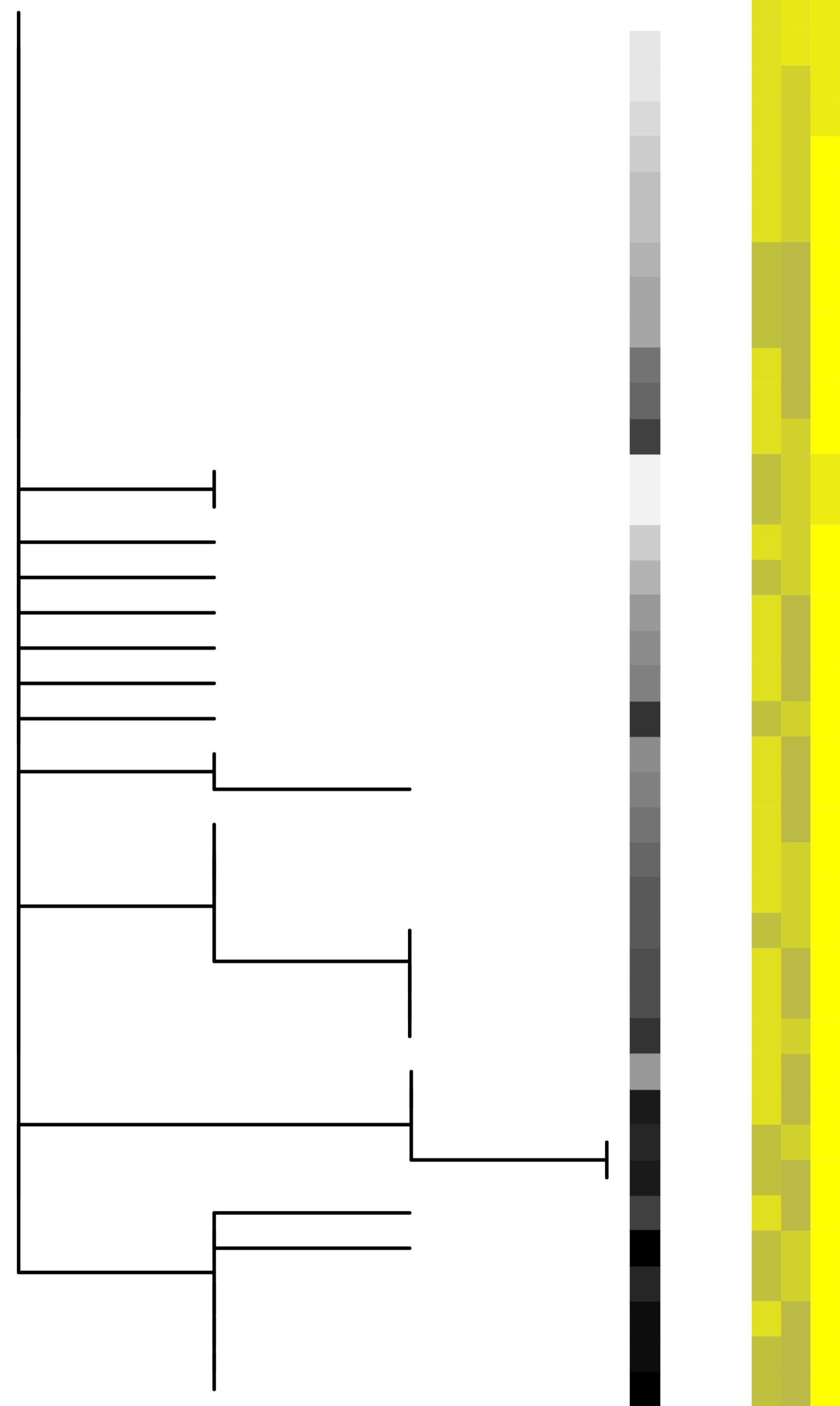

B

Tree scale: 0.0001

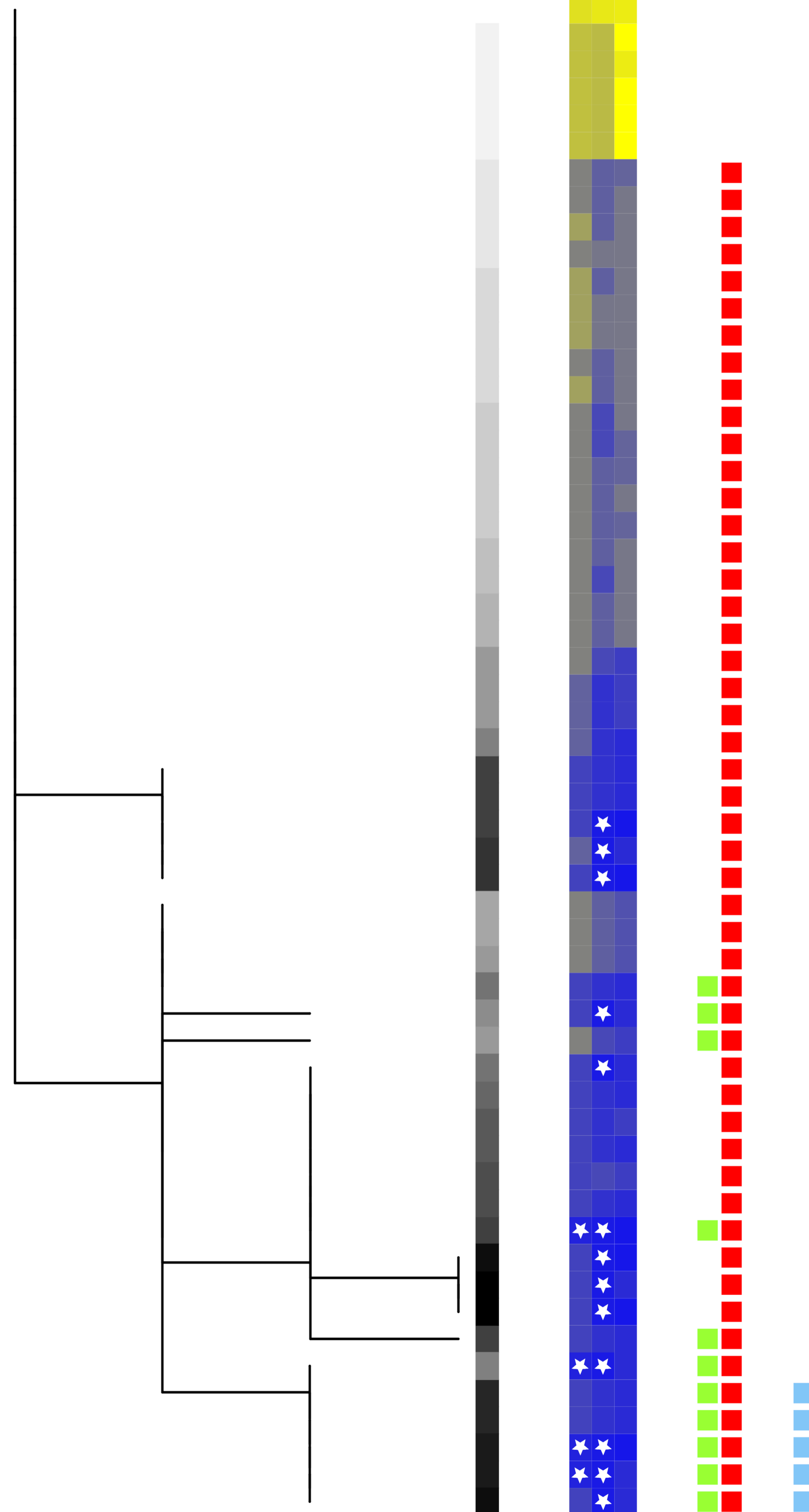

C

Tree scale: 0.001

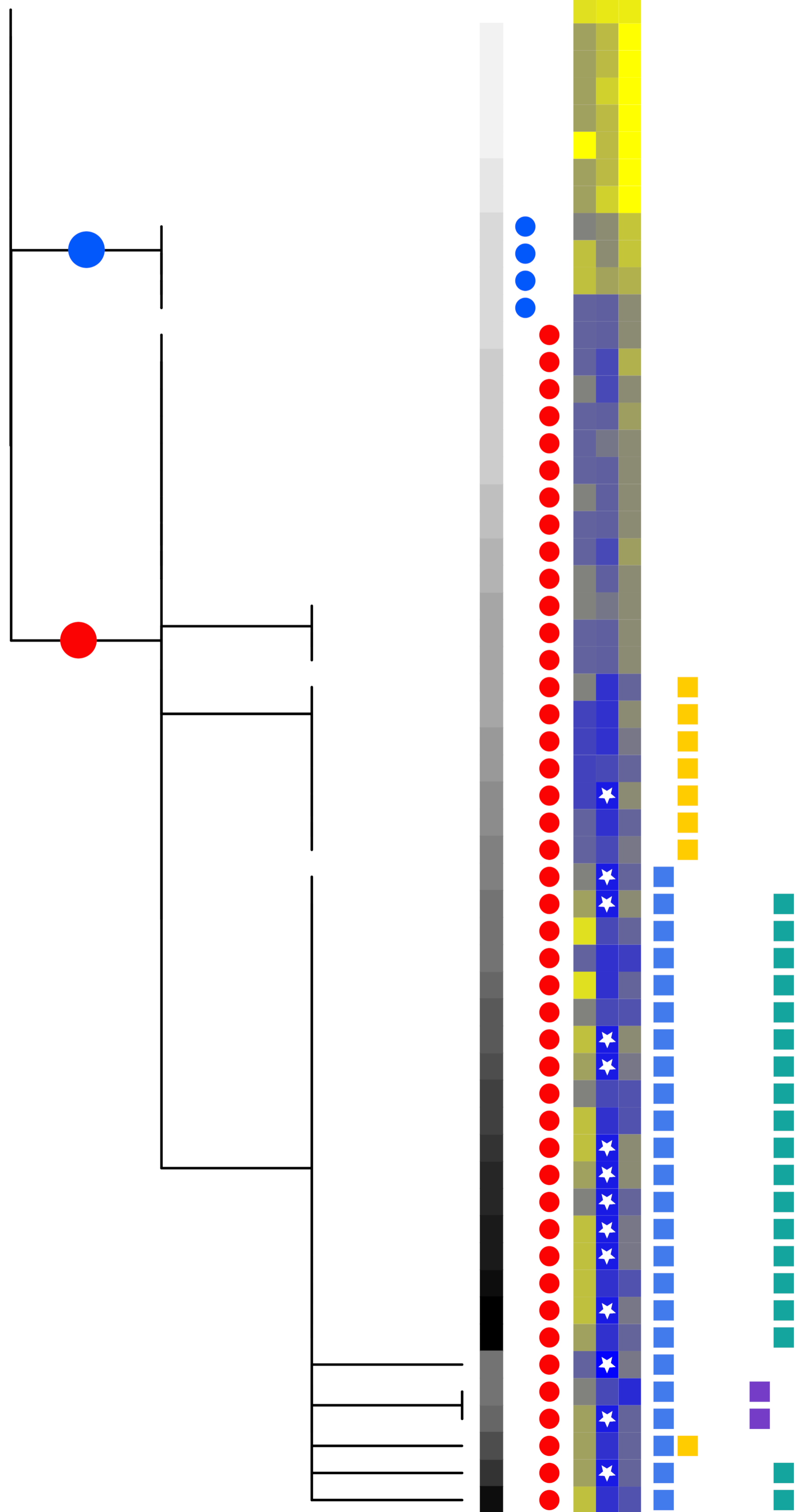

D

Tree scale: 0.0001

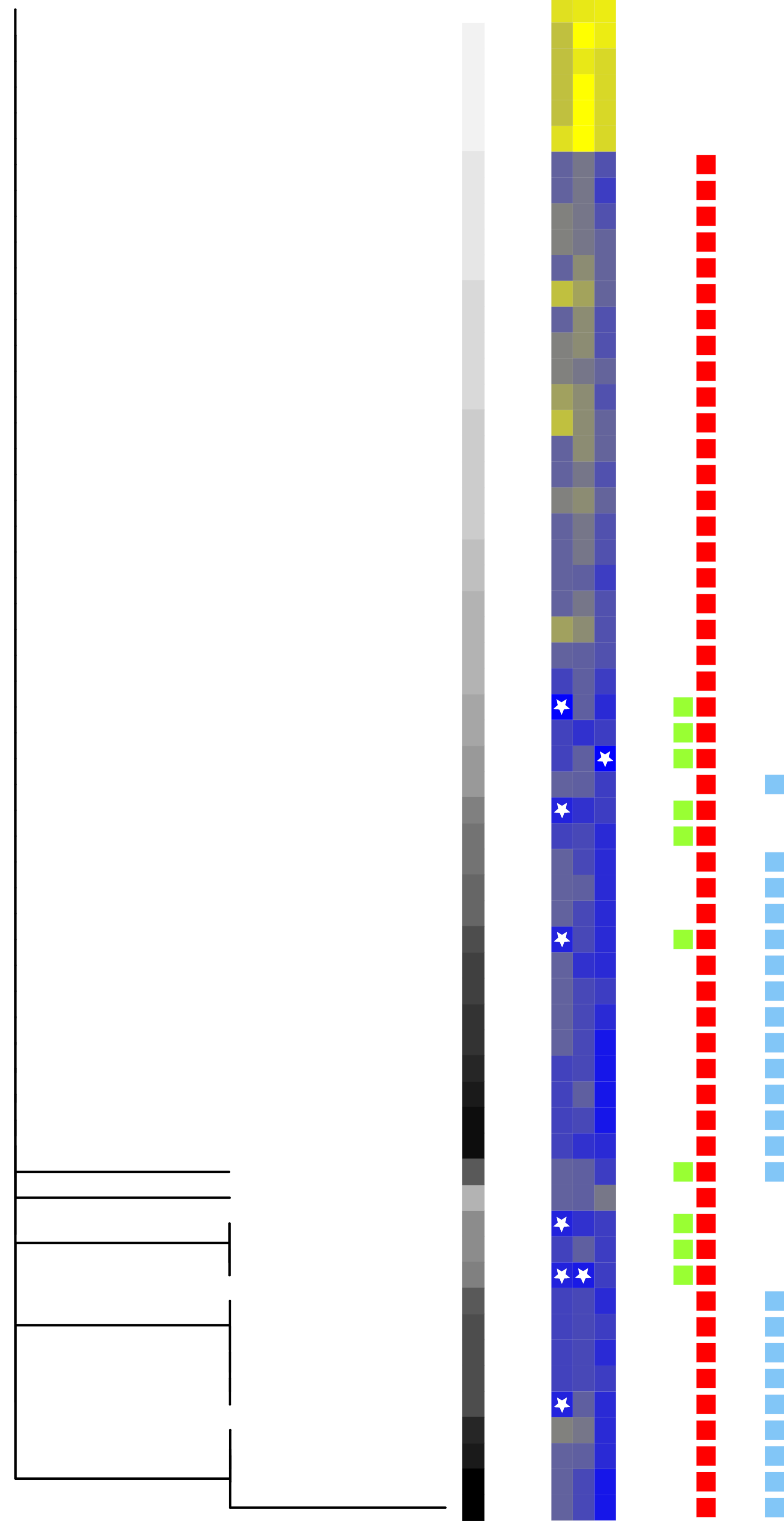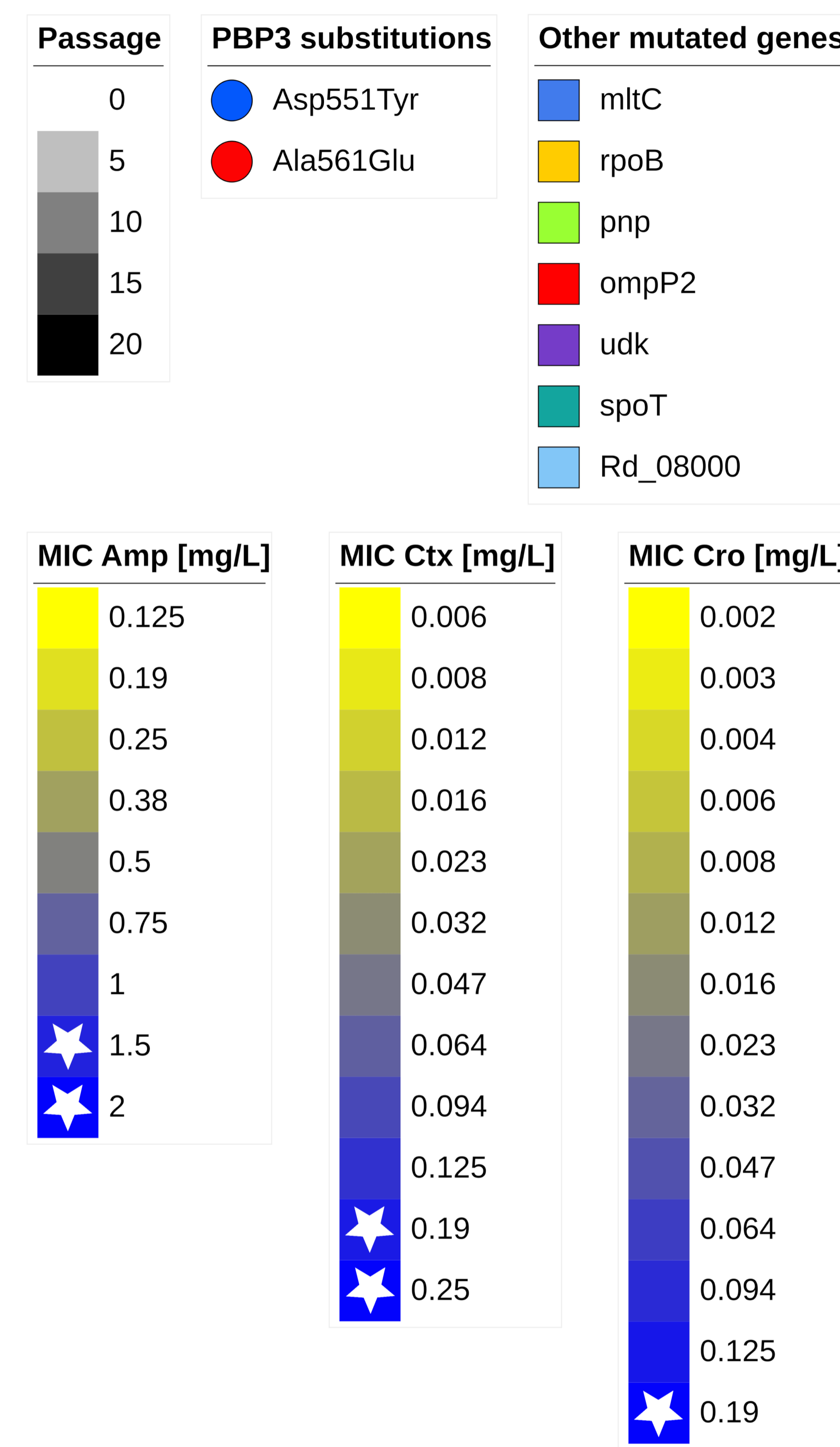

Supplement: Fig. S3 — Maximum likelihood phylogeny of H. influenzae Rd KW20 clones evolved in the absence of an antibiotic pressure and in three replicate populations evolved in cefotaxime. [file aac.00576-25-s0003.pdf]

**A**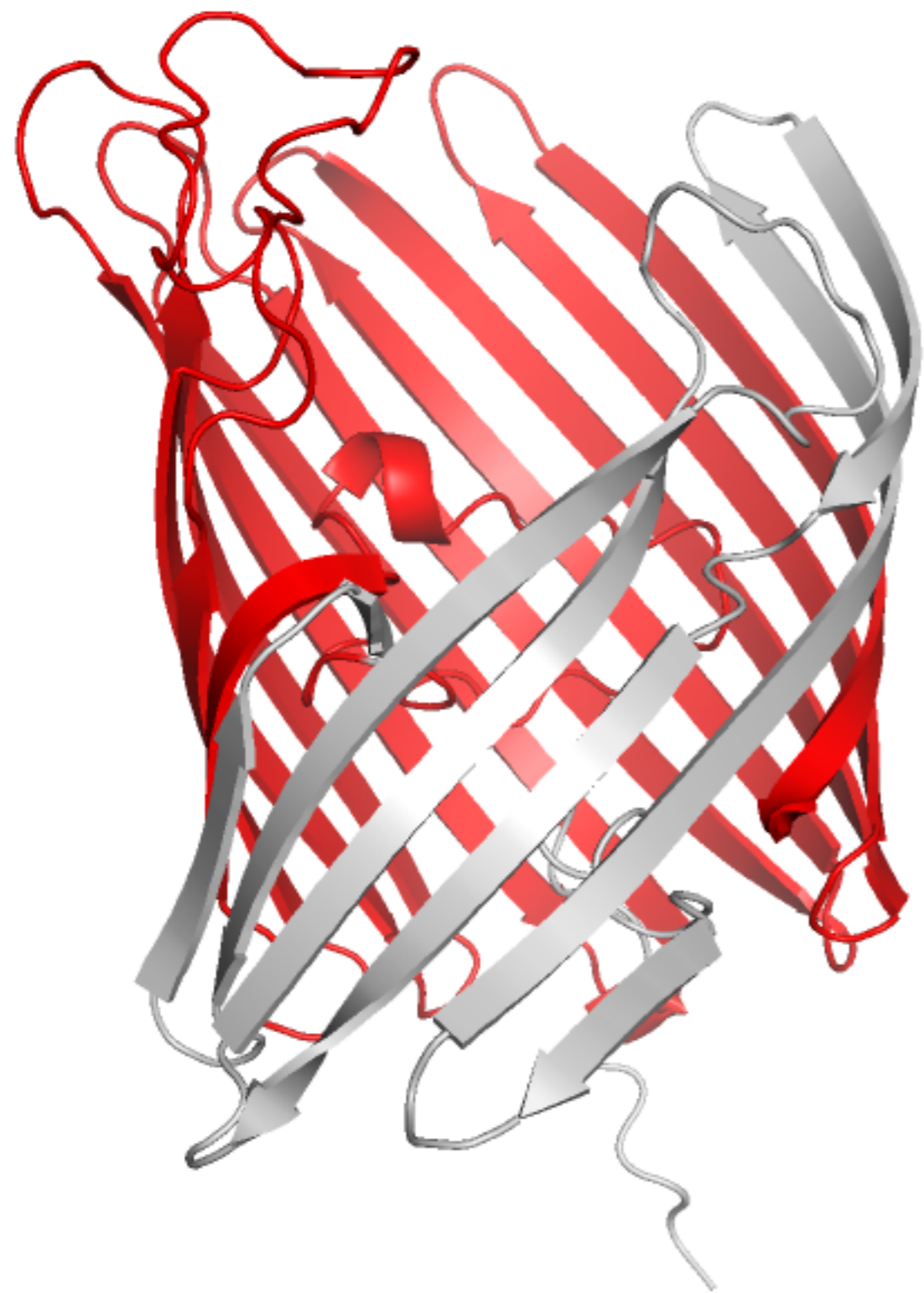**B**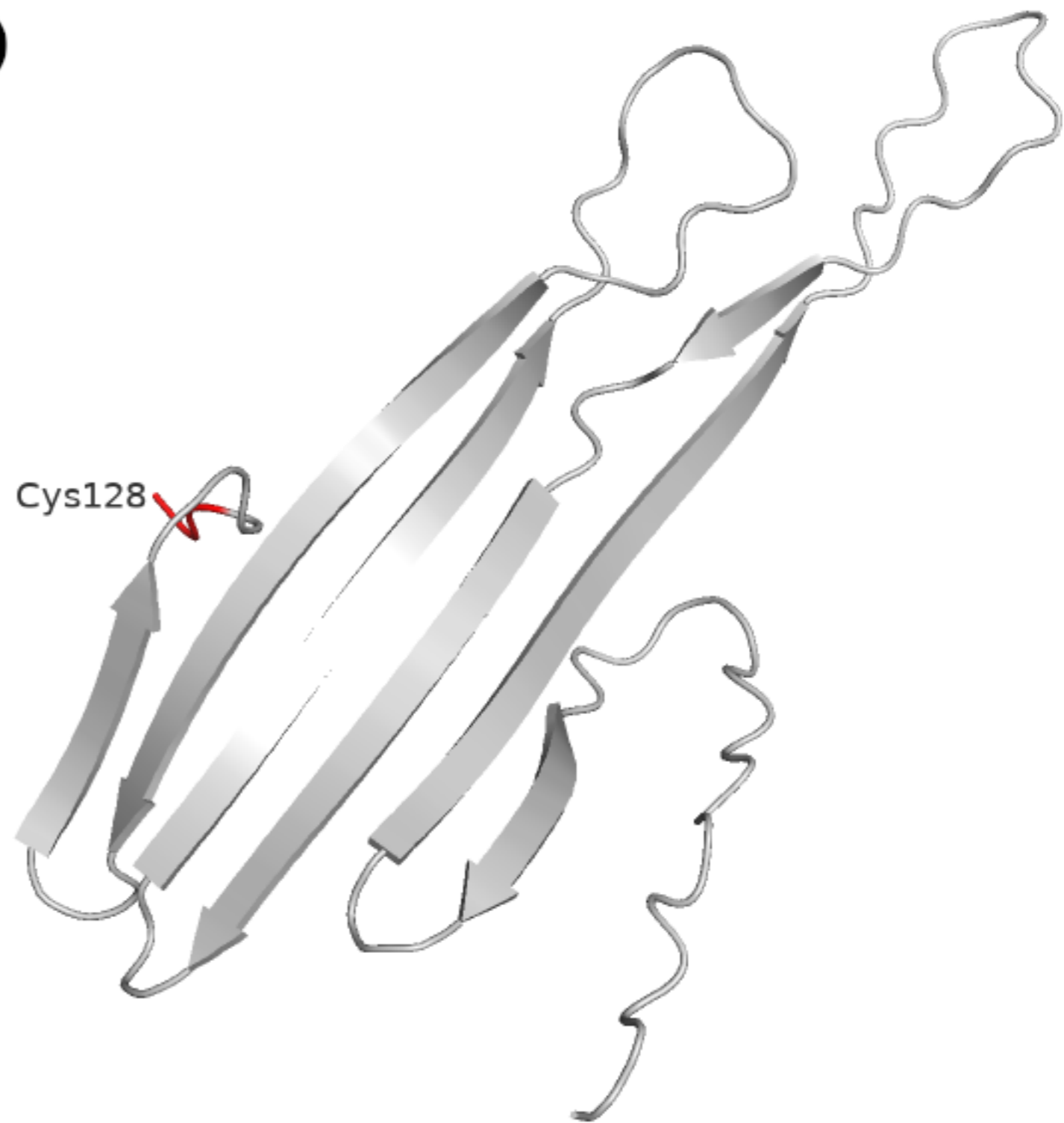

Supplement: Fig. S4 — Crystal structure of outer membrane protein P2 of wild-type H. influenzae Rd KW20 (A) and mutated OmpP2 (B). [file aac.00576-25-s0004.pdf]

**A**

wild type

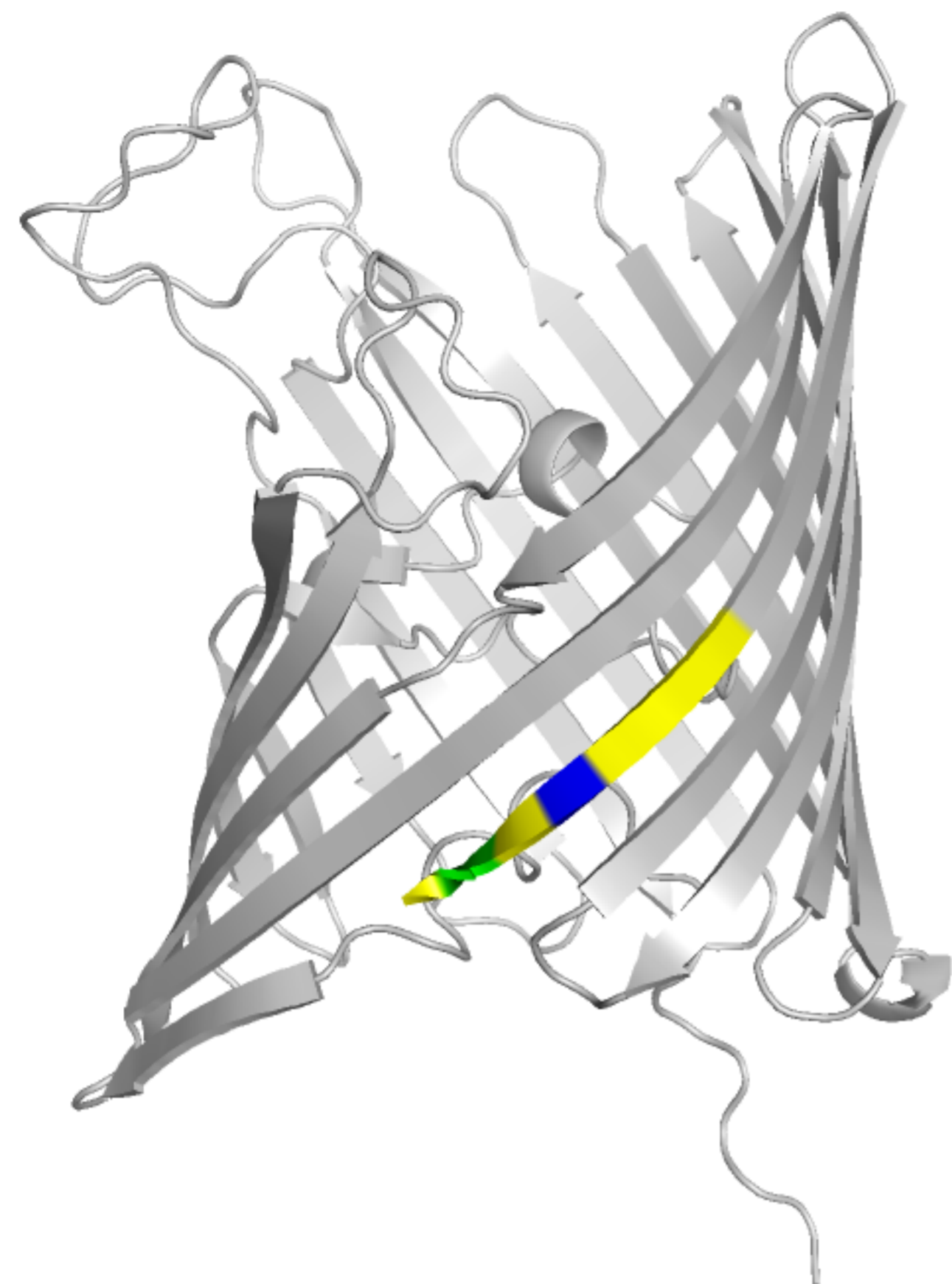**B**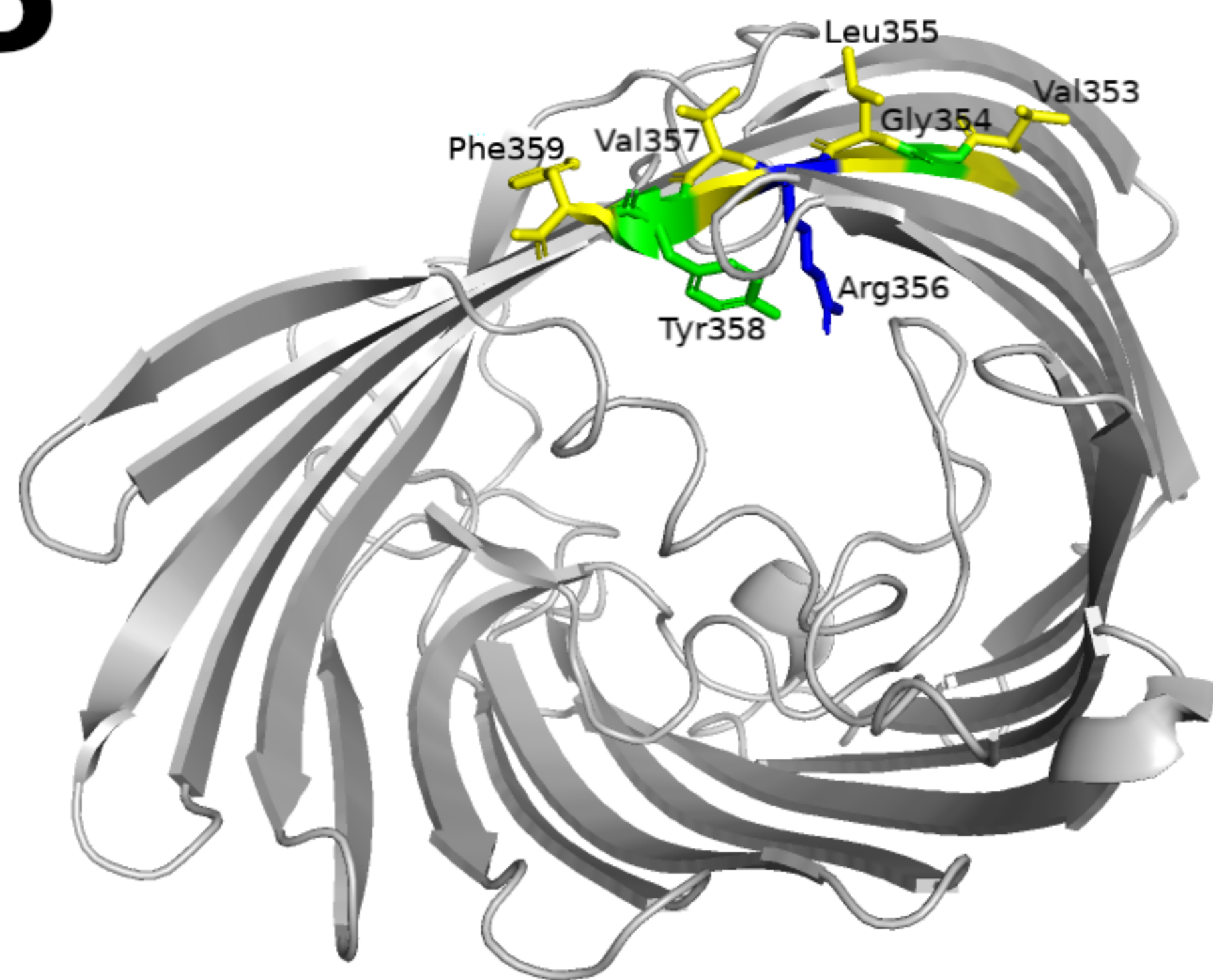**C**

inversion

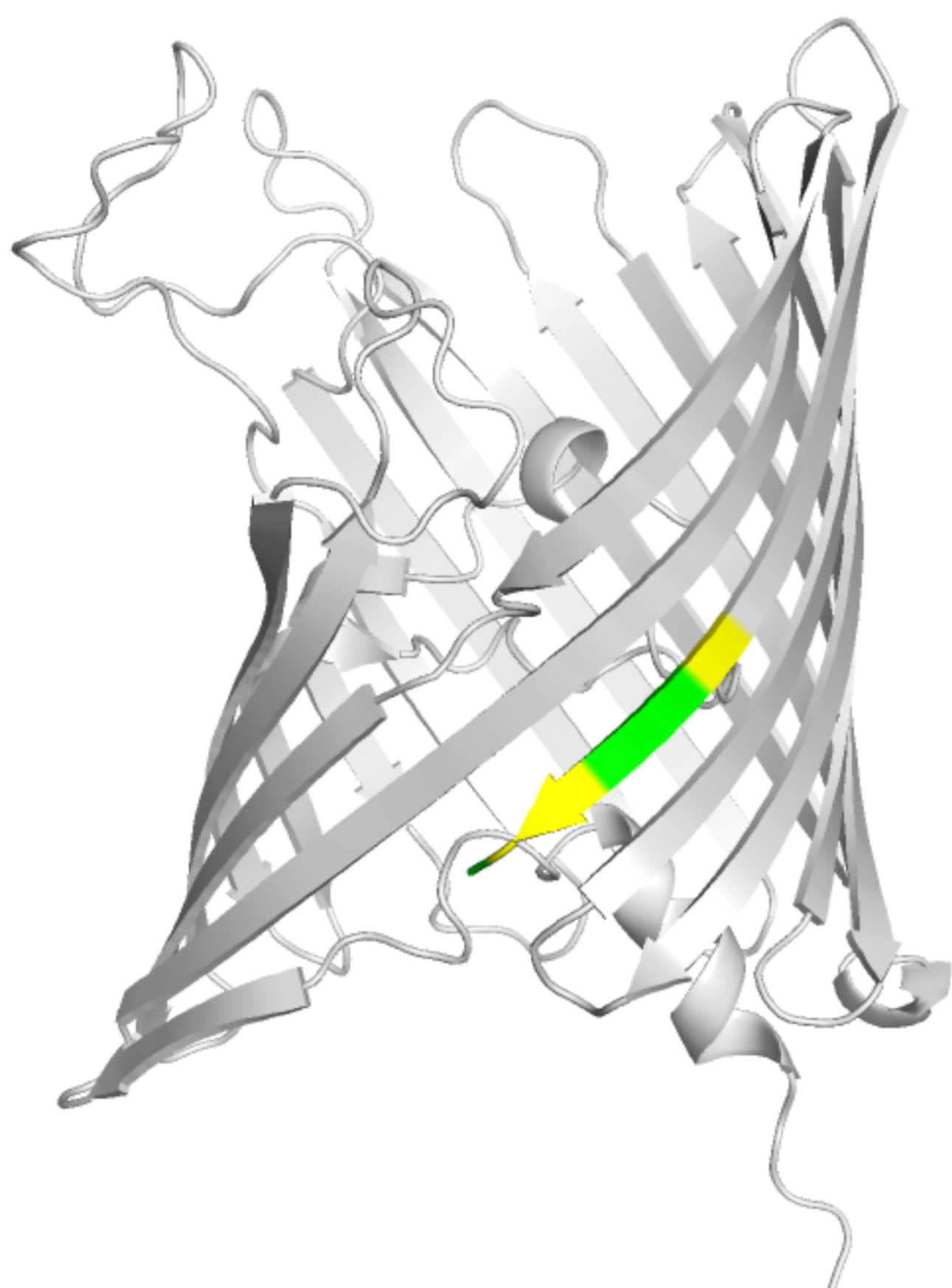**D**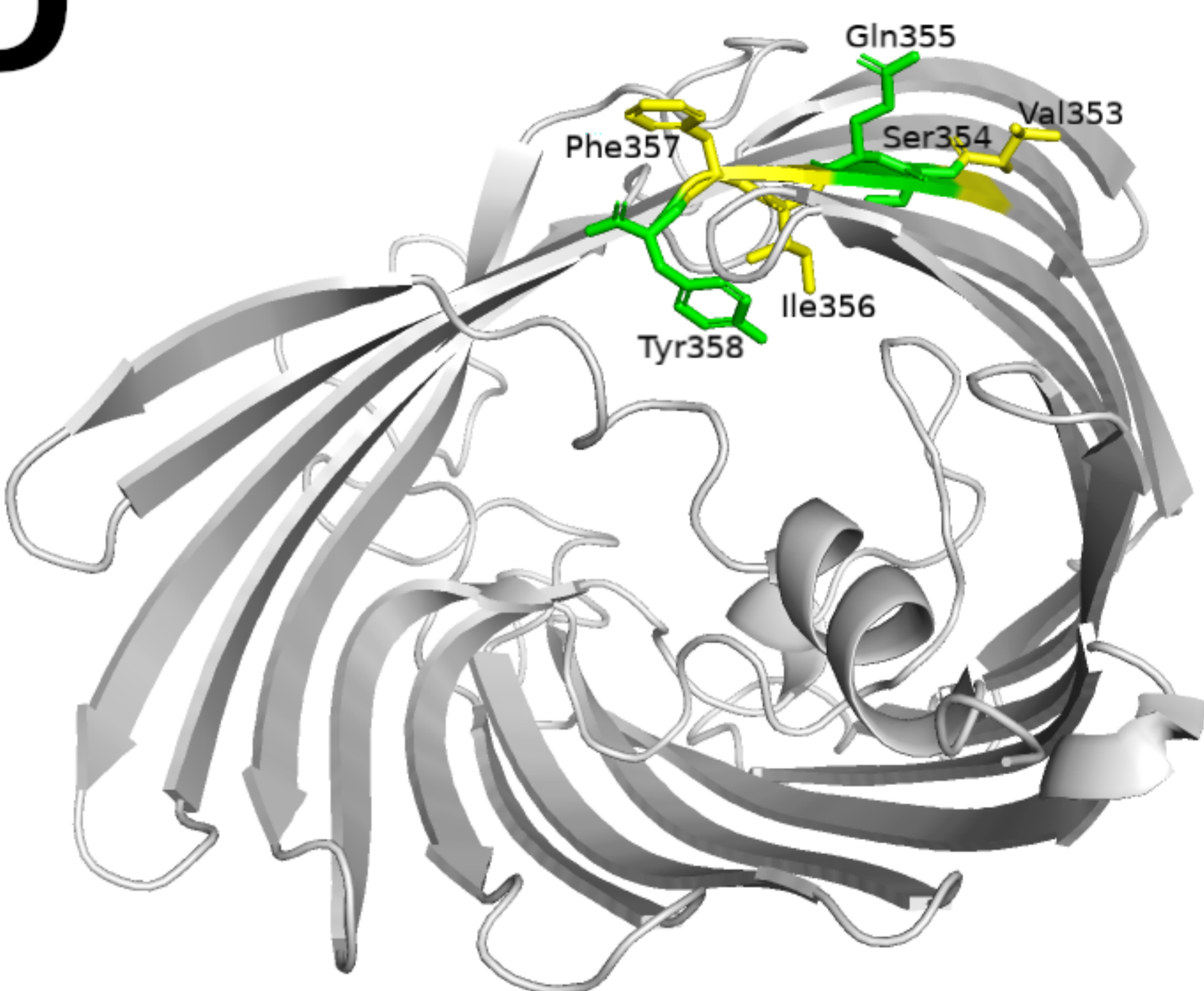**E**

deletion

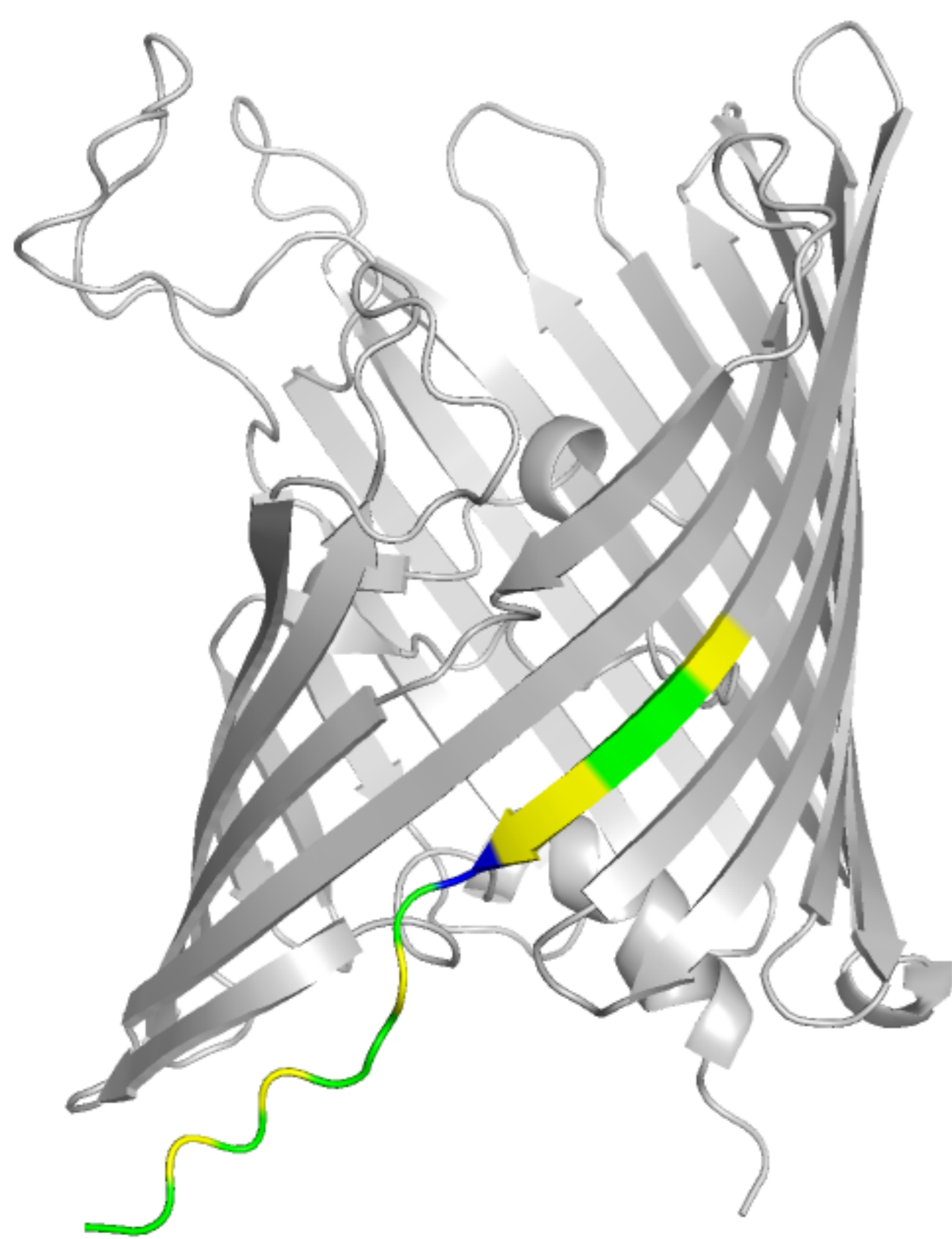**F**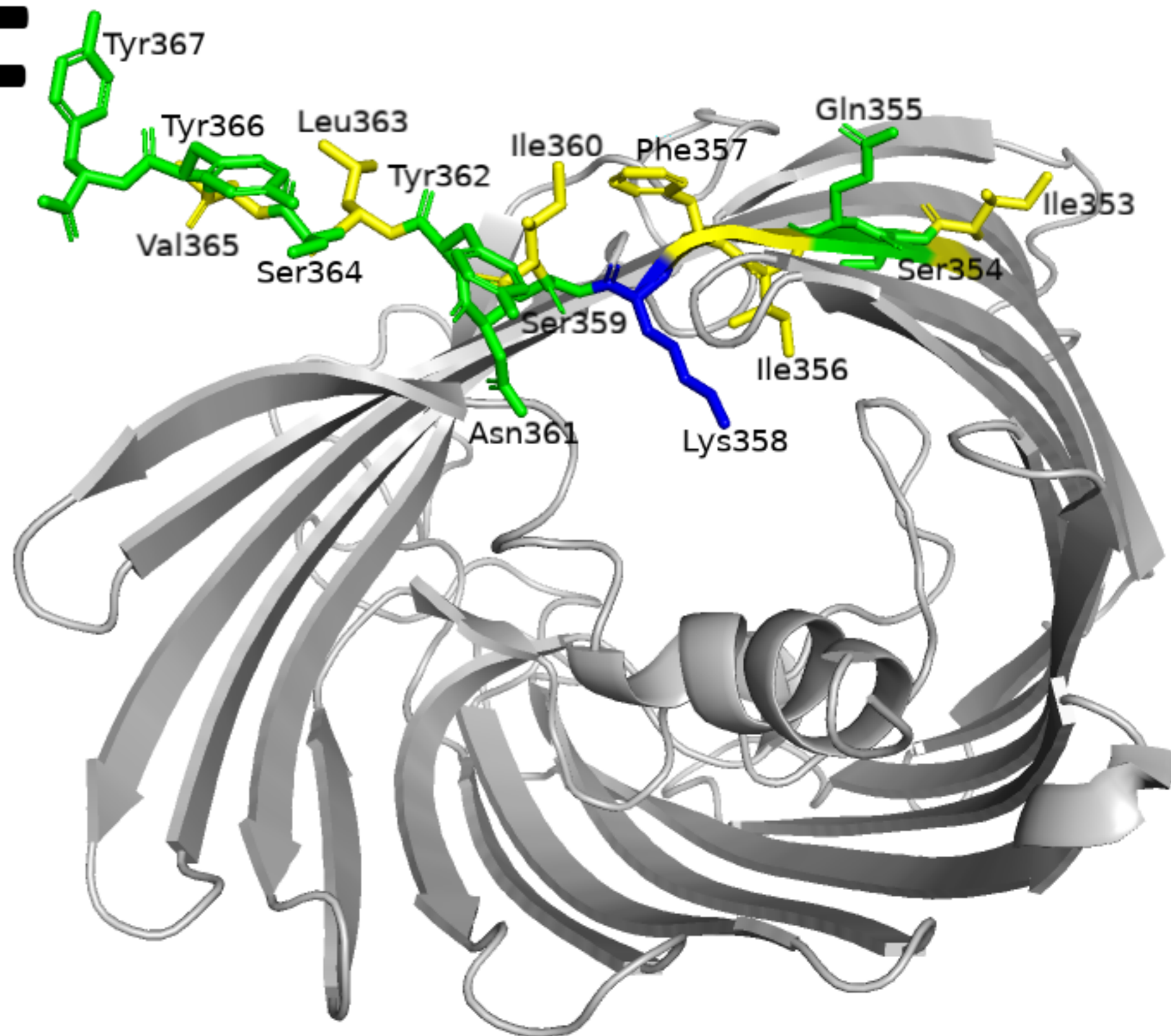

Supplement: Fig. S5 — Crystal structure of outer membrane protein P2 of wild-type H. influenzae Rd KW20, shown in side view (A) and bottom view (B) and two variants of OmpP2. [file aac.00576-25-s0005.pdf]

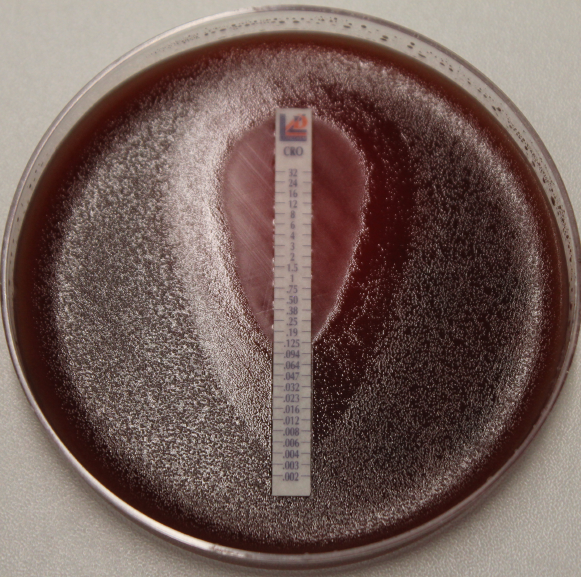

Supplement: Fig. S6 — Gradient diffusion test demonstrating heteroresistance. [file aac.00576-25-s0006.png]
