# Supplementary material for: Evolution of beta-lactam resistance causes fitness reductions and several cases of collateral sensitivities in the human pathogen Haemophilus influenzae
Source: Antimicrob Agents Chemother. 2025 Sep 22;69(11):e00576-25. doi: 10.1128/aac.00576-25 (PMC12587571; doi:10.1128/aac.00576-25)
Supplement: Supplemental material — Captions for all supplemental figures. [file aac.00576-25-s0007.docx]

## Supplementary Figures

Supplementary Figure S1: Graphical protocol of the performed multi-step evolution experiment (generated with BioRender).

Supplementary Figure S2: Graphical protocol of the performed competitive growth assay (generated with BioRender).

Supplementary Figure S3: Maximum likelihood phylogeny of *H. influenzae* Rd KW20 clones evolved in the absence of an antibiotic pressure (A, 40 clones) and in three replicate populations evolved in ceftriaxone (B, replicate 1 with 57 clones; C replicate 2 with 56 clones; D, replicate 3 with 59 clones). Amino acid substitutions in the penicillin binding protein 3 (PBP3) and presence of mutations in specified genes as well as minimum inhibitory concentration (MIC) changes over time are color coded. Strains that exceeded the clinical breakpoint for ampicillin/cefotaxime/ceftriaxone according to EUCAST breakpoints are marked with a white star at the MIC heatmap.

The specified genes are those in which at least 20 clones from the whole set of selected clones exhibited mutations but did not mutate under antibiotic-free conditions.

Supplementary Figure S4: Crystal structure of Outer membrane protein P2 of wild-type *H. influenzae* Rd KW20 (A) and mutated OmpP2 (B) truncated due to p.Thr126fs (c.376_377insT). Structures were predicted using the AlphaFold Server powerd by AlphaFold 3 (26) and visualized using PyMOL v3.0. Deviations between the two variants are highlighted in red.

Supplementary Figure S5: Crystal structure of Outer membrane protein P2 of wild-type *H. influenzae* Rd KW20, shown in side view (A) and bottom view (B) and two variants of OmpP2 altered due to p.Gly354fs (C and D, inversion) and p.Val353fs (E and F, deletion). Structures were predicted using the AlphaFold Server powerd by AlphaFold 3 (26) and visualized using PyMOL v3.0. Deviations between the two variants are labeled and highlighted in yellow (hydrophobic), green (polar uncharged), and blue (positively charged).

Supplementary Figure S6: Gradient diffusion test demonstrating phenotypic heterogeneity. The image shows two distinct inhibition zones surrounding the antibiotic gradient strip, indicative of a heterogeneous bacterial population. The inner zone corresponds to a subpopulation with higher antibiotic resistance, while the outer zone represents the more susceptible majority.
